# Prevalence of depressive symptoms among medical students in Pakistan: a systematic review and meta-analysis

**DOI:** 10.1136/bmjopen-2026-116544

**Published:** 2026-06-22

**Authors:** Hamideh Ebrahimi, Rashida Bibi, Mohammad Hossein Falahatpisheh, Dr Sundeep Chaitanya Vedithi

**Affiliations:** 1 Lahore School of Nursing, The University of Lahore, Lahore, Punjab, Pakistan; 2 Farkhanda Institute of Nursing and Public Health, Gandhara University, Peshawar, Khyber Pakhtunkhwa, Pakistan; 3 Nursing, McGill University, Montreal, Quebec, Canada; 4 Medicine, University of Cambridge School of Clinical Medicine, Cambridge, Cambridgeshire, UK

**Keywords:** Systematic Review, Meta-Analysis, Depression & mood disorders, Schools, Medical, Prevalence

## Abstract

**Abstract:**

**Objectives:**

Depressive symptoms are highly prevalent among medical students globally, yet estimates from Pakistan remain fragmented and inconsistent across studies. Medical students in Pakistan face multiple academic, social and environmental stressors that may increase their risk of depression. We conducted a systematic review and meta-analysis to estimate the prevalence of depressive symptoms among medical students in Pakistan and to explore sources of heterogeneity across studies.

**Design:**

Systematic review and meta-analysis reported in accordance with the Meta-analysis Of Observational Studies in Epidemiology (MOOSE) guidelines.

**Data sources:**

MEDLINE (via PubMed), Web of Science, Embase, Scopus, APA PsycInfo and Pakistani journals were searched without date restriction up to September 2025.

**Eligibility criteria:**

Observational studies conducted among medical students in Pakistan reporting prevalence of depressive symptoms using validated assessment tools (eg, PHQ-9, BDI, DASS-21) were included.

**Data extraction and synthesis:**

Two independent reviewers screened studies, extracted data and assessed methodological quality using the Joanna Briggs Institute tool. Random-effects meta-analysis was conducted. Subgroup analyses (province and assessment instrument) and meta-regression (publication year) were performed. Heterogeneity and publication bias were assessed using I² statistics, funnel plots and Egger’s and Begg’s tests.

**Results:**

Seventy-one studies contributed 72 prevalence estimates, including approximately 23 000 medical students. The reported prevalence of depressive symptoms ranged from 9% to 94%, with considerable heterogeneity across studies (I²=99.15%, p<0.001). Subgroup analysis showed slightly higher prevalence estimates in Sindh compared with Punjab and other regions; however, subgroup differences were not statistically significant (Q=1.00, df=2, p=0.61). Prevalence estimates also varied according to the assessment instrument, with generally higher estimates reported using PHQ-9 and DASS-21. Meta-regression demonstrated a significant positive association between publication year and reported prevalence (β=0.016, SE=0.007, 95% CI 0.002 to 0.031, p=0.03). Evidence of publication bias was identified by Egger’s test (p=0.024) and Begg’s test (p<0.001), although adjusted analyses yielded broadly consistent findings.

**Conclusions:**

The prevalence of depressive symptoms among medical students in Pakistan varies widely across studies, with evidence suggesting an increasing trend over time. However, the findings should be interpreted with caution due to substantial heterogeneity across studies, variability in assessment instruments and cut-off thresholds, and methodological limitations in some included studies. Despite these limitations, the findings highlight the need for scalable mental health interventions, including screening, counselling and educational reforms.

**PROSPERO registration number:**

CRD420251160506.

**STRENGTHS AND LIMITATIONS OF THIS STUDY**

- This study employed a comprehensive search strategy across multiple international databases and local Pakistani journals without date restriction.
- Study selection, data extraction and quality assessment were conducted independently by two reviewers using standardised methods.
- The methodological quality of included studies was assessed using the Joanna Briggs Institute critical appraisal tool.
- Variation in study designs and assessment instruments across included studies may have introduced methodological heterogeneity.
- Differences in diagnostic criteria and measurement thresholds across studies may have affected the comparability of prevalence estimates.

## Introduction

 Depression is a common mental disorder worldwide, characterised by persistent sadness, loss of interest or pleasure, low energy, sleep disturbances and impaired concentration.^1^ Beyond its impact on emotional well-being, depression can adversely affect cognitive functioning, academic performance and overall quality of life, even in its milder forms.^2^ Medical students are particularly affected, with global evidence indicating a high prevalence of depressive symptoms; for example, a landmark study published in JAMA in 2016 reported that approximately 27% of medical students experience depression or depressive symptoms.^3^ This elevated burden has been linked to academic pressures, demanding workloads, long clinical hours and frequent exposure to patients’ suffering.^4^ Moreover, evidence from specific regions suggests even higher prevalence rates, with a recent review of studies from the Arab region reporting estimates ranging from 40% to 77.9%.^5^

In Pakistan, several individual studies have reported high levels of depression and anxiety among medical and health sciences students. For instance, a study conducted in Karachi found that 43.89% of medical students experienced depression and anxiety.^6^ Furthermore, a recent systematic review and meta-analysis reported that 42.66% of university students in Pakistan experience depressive symptoms, highlighting a substantial mental health burden in this population.^7^ However, this study included students from diverse academic disciplines and did not provide a focused synthesis for medical students, who are exposed to distinct academic and clinical stressors.

Medical students in Pakistan are exposed to additional challenges, including limited availability of mental health services, stigma associated with psychological disorders, financial constraints and restricted access to professional psychological support.^6 8^ These contextual factors may further exacerbate the risk and severity of depression in this group.

Despite growing evidence indicating a high prevalence of depressive symptoms among medical students in Pakistan, several important gaps remain. Existing studies show considerable variability in prevalence estimates, likely due to differences in study design, assessment tools and sample characteristics. Moreover, there has been no comprehensive and up-to-date systematic synthesis focusing specifically on medical students in Pakistan, with detailed subgroup analyses (eg, by geographic region, assessment instrument and temporal trends) and rigorous evaluation of methodological quality and risk of bias.

Therefore, this systematic review and meta-analysis aimed to estimate the prevalence of depressive symptoms among medical students in Pakistan and to explore sources of heterogeneity across studies. In addition, we sought to examine variations in prevalence based on geographic region, assessment tools and publication year, while also assessing the methodological quality of included studies.

## Method

This systematic review and meta-analysis was reported in accordance with the Meta-analysis Of Observational Studies in Epidemiology (MOOSE)^9^ and was registered in the PROSPERO database with registration number CRD420251160506. A thorough search strategy was implemented across various international databases, including MEDLINE (via PubMed), Web of Science Core Collection, Embase, Scopus and APA PsycInfo. Manual searches were also conducted on scientific websites and Pakistani research journals, such as the Pakistan Journal of Medical Sciences, Journal of the Pakistan Medical Association and PakMediNet, to ensure all relevant studies were included.

The search utilised a mix of controlled vocabulary and free-text terms associated with depression, medical students, medical education and Pakistan. Key terms included depress, (medic AND)student* OR trainee* OR learner*((, resident, “doctor in training”, “physician in training”*, and Pakistan. Logical operators AND and OR were used to combine the search terms to encompass all potentially relevant studies. The search was not limited by time, and it was finalised in September 2025. A comprehensive outline of the search strategy can be found in online supplemental file 1).

The PICO framework was used to determine the eligibility criteria and formulate the research question:

Population (P): medical students in Pakistan.

Intervention/exposure (I): studies that reported the mean score or prevalence (%) of depressive symptoms.

Comparison (C): in most cases, a comparison group was not applicable for this type of study, except when data compared pre and post-COVID-19 periods.

Outcome (O): the prevalence and severity of depressive symptoms, measured using validated tools like the PHQ-9, BDI, DASS-21 and other standardised instruments.

### Inclusion criteria

- The study focused on medical students in Pakistan.
- The study investigated the prevalence or incidence of depressive symptoms in this population.
- Depression was assessed using validated measurement tools like PHQ-9, BDI, DASS-21 or CES-D and other standardised instruments.
- Articles were published in English or Urdu.
- The study design was cross-sectional or another observational design that provided prevalence data.

### Exclusion criteria

- The study did not involve medical students, including students from allied health sciences or general university students.
- Studies that combined depression scores with anxiety or stress without separate depression results.
- Narrative reviews, letters, systematic or literature reviews, case reports or studies without quantitative data.
- Studies that lacked sufficient data to calculate depression prevalence.
- Unpublished articles or studies with inaccessible full-text, even after contacting the corresponding author.

### Study selection process

**Figure 1:**
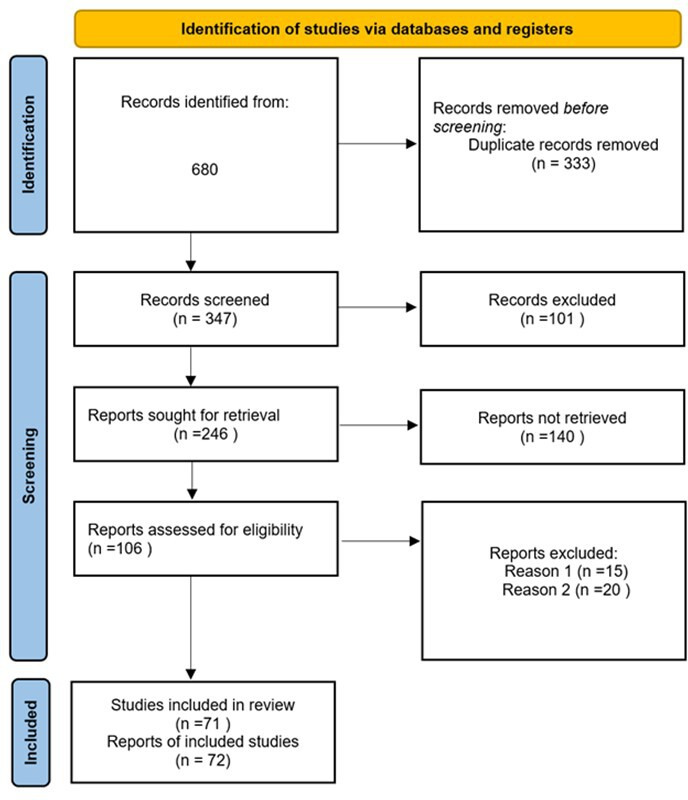
PRISMA flow diagram. PRISMA, Preferred Reporting Items for Systematic Reviews and Meta-Analyses.

All search results were imported into EndNote software, and duplicate records were eliminated. The screening process was carried out in two phases. Initially, two researchers independently reviewed the titles and abstracts of all retrieved studies to evaluate their relevance. Subsequently, the full texts of potentially eligible studies were scrutinised for final inclusion. Any discrepancies between reviewers were resolved through discussion and, if needed, by consulting a third reviewer. The study selection process is depicted in the Preferred Reporting Items for Systematic Reviews and Meta-Analyses (PRISMA) flow diagram (figure 1).

### Data extraction

Two reviewers independently extracted data from each included study. The extracted information included the author’s name, publication year, geographical region, total sample size, sample size by gender (male and female), depression assessment tool, cut-off point, depression prevalence (%) and study quality. Any discrepancies in the extracted data were resolved through discussion and consensus.

### Depression assessment instruments

Depression was evaluated in the studies using various validated assessment tools, such as the Depression Anxiety Stress Scale (DASS-21 and DASS-42),^10^ Beck Depression Inventory I and II (BDI-I, BDI-II),^11 12^ Zung Self-Rating Depression Scale (ZUNG),^13^ Patient Health Questionnaire (PHQ-9 and PHQ-2),^14^ Burns Depression Checklist,^15^ Hamilton Depression Rating Scale (HAM-D),^16^ Hospital Anxiety and Depression Scale (HADS)^17^ and Kutcher Adolescent Depression Scale (KADS).^18^

To ensure consistency and comparability, prevalence estimates were extracted based on the cut-off definitions reported in each individual study and interpreted with reference to the validated thresholds of the respective assessment instruments. These thresholds generally corresponded to established categories of symptom severity (eg, mild, moderate or severe), although the specific cut-off points and classification of depression varied across instruments. For example, instruments such as the PHQ-9 and DASS-21 use standardised score ranges to categorise severity levels, while tools like the BDI and HADS apply different threshold definitions. As a result, the operational definition of depression was not uniform across studies, particularly regarding whether mild symptom thresholds were classified as depression, which may have introduced some variability in classification.

### Data analysis

A random-effects meta-analysis was conducted using the DerSimonian and Laird method. All statistical analyses were performed using STATA software V.17. Heterogeneity among studies was assessed using Cochran’s Q test and the I² statistic. The interpretation of I² followed conventional thresholds: low (<25%), moderate (25%–50%), substantial (50%–75%) and considerable (>75%) heterogeneity. Forest plots were used to display individual study estimates with 95% CIs. Proportions were transformed using the Freeman–Tukey double arcsine transformation to stabilise variance and account for variability in prevalence estimates across studies.

Subgroup analyses and meta-regression were performed to explore potential sources of heterogeneity. Prevalence estimates were extracted based on the cut-off definitions reported in the original studies and interpreted with reference to the validated thresholds of the respective instruments. Due to variation in the operational definitions of depression across studies, full standardisation was not possible.

Subgroup analyses were conducted according to geographical region (province or area within Pakistan) and assessment instrument. Meta-regression analysis was performed using a random-effects model to examine the association between publication year and reported prevalence. A univariable model was fitted using restricted maximum likelihood (REML). Regression coefficients (β), standard errors (SE), 95% CIs and p values were reported. Residual heterogeneity was assessed using the I² statistic.

Publication bias was assessed through visual inspection of funnel plots and Egger’s test.

### Quality assessment

The methodological quality of the included studies was assessed using the Joanna Briggs Institute (JBI) Critical Appraisal Checklist for Studies Reporting Prevalence Data. This standardised tool consists of nine items evaluating key aspects of methodological rigour, including sampling methods, sample size adequacy, data analysis, validity and reliability of measurement, and response rate.

Each item was rated as ‘Yes’, ‘No’, ‘Unclear’ or ‘Not applicable’. A score of 1 was assigned to ‘Yes’ responses, while all other responses received a score of 0. The total quality score for each study ranged from 0 to 9. Based on the total score, studies were categorised into three levels of methodological quality: low quality (≤3), moderate quality (4–6) and high quality (7–9).^19^

### Patient and public involvement

Patients and/or the public were not involved in the design, conduct, reporting or dissemination plans of this research.

## Results

### Systematic review

A total of 71 studies that met the inclusion criteria were included in this systematic review, resulting in 72 prevalence estimates of depression. The discrepancy between the number of studies and prevalence estimates was due to one study that evaluated depression at two distinct time points before and after the COVID-19 pandemic and reported each estimate separately.^20^

The Depression Anxiety Stress Scale (DASS-21) was the most commonly used instrument for assessing depression in the included studies, utilised in 30.6% of the studies, followed by the Patient Health Questionnaire (PHQ-9) in 20.8% and the Hospital Anxiety and Depression Scale (HADS) in 11.1%. Other instruments such as the Beck Depression Inventory I and II (BDI-I, BDI-II), Zung Self-Rating Depression Scale (ZUNG), CES-D10 and DASS-42 were less frequently employed. The average sample size across the studies was 325 participants (ranging from 66 to 2270), indicating significant variability in study size and geographic representation across Pakistan. The province of Punjab accounted for the highest number of reports (37 studies; 52.1%), followed by Sindh (20 studies; 28.2%). The remaining studies were conducted in Khyber Pakhtunkhwa (5.6%), Islamabad (2.8%), Kashmir (2.8%) and in multicentre or semimulticentre settings (four studies; 5.6%). The characteristics of the included studies are summarised in online supplemental table 1.

### Meta-analysis

Although a random-effects model was applied, the prevalence estimates varied widely across studies (9% to 94%), with substantial heterogeneity (I² = 99.15%), suggesting that a single pooled estimate may not adequately summarise the findings.

**Figure 2:**
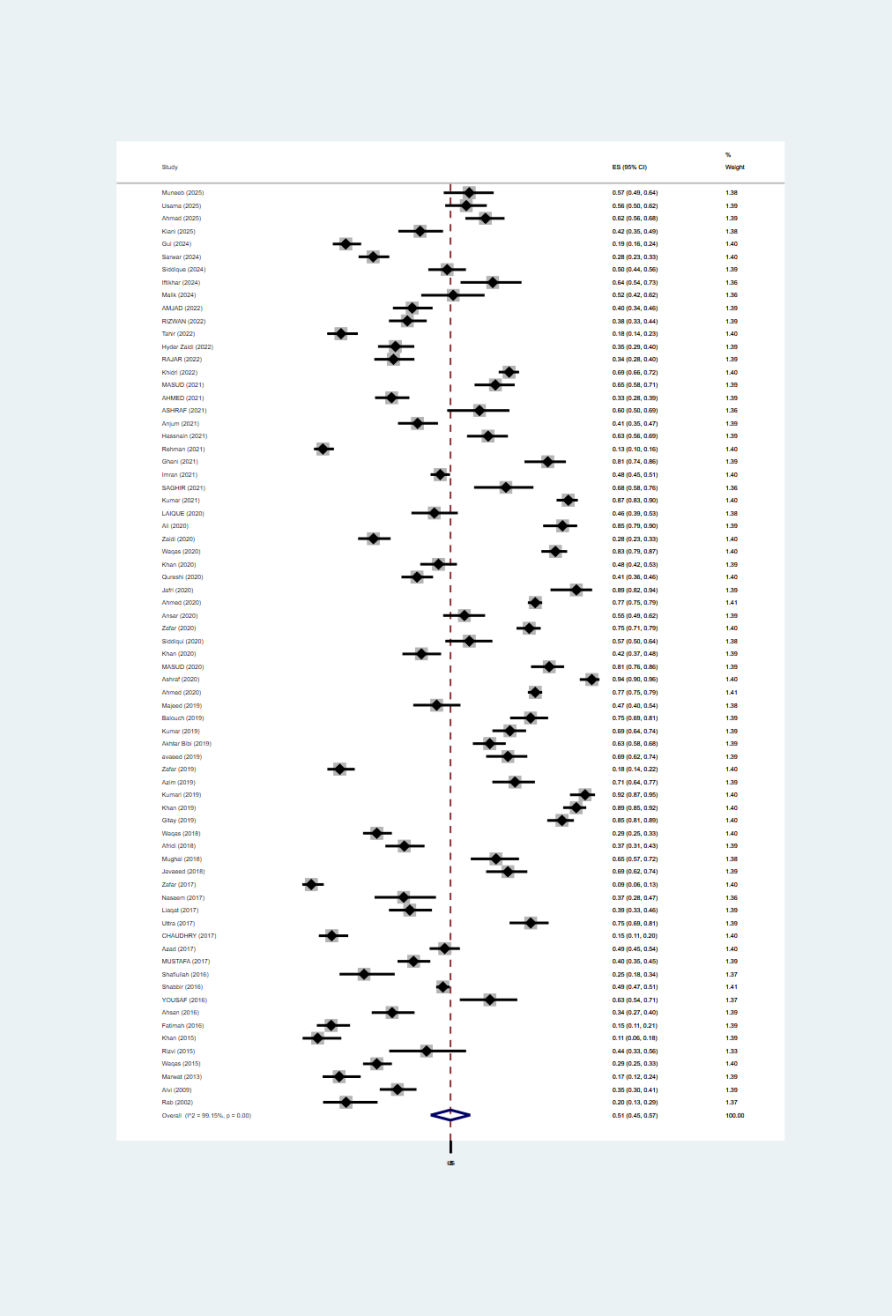
Prevalence of depression in the included studies (forest plot).

Significant heterogeneity was observed among the studies included (I²=99.15%, τ²=0.07, p<0.001), indicating a wide range of prevalence estimates across studies. Reported rates of depression varied from 9% to 94%, reflecting differences in assessment tools, locations, study periods and academic settings. Figure 2 displays the forest plot showing the prevalence of depressive symptoms in the included studies.

### Subgroup analysis by province

The subgroup analysis based on provincial distribution showed substantial variability in prevalence estimates across regions. In Sindh, reported prevalence ranged from approximately 9% to 92%, with generally higher estimates compared with other regions. In Punjab, prevalence estimates also varied widely, ranging from about 10.9% to 94%. Studies conducted in other regions, including Khyber Pakhtunkhwa, Islamabad and Kashmir, reported prevalence estimates ranging from approximately 12% to 68%.

Significant heterogeneity was observed within all three subgroups (Sindh: I²=99.4%, p<0.001; Punjab: I²=99.1%, p<0.001; Others: I²=97.7%, p<0.001), indicating considerable variability within each region. The test for subgroup differences indicated that the variation in depression prevalence across provinces was not statistically significant (Q=1.00, df=2, p=0.61).

Overall, these findings suggest that while prevalence estimates differ across provinces, a consistently high and variable burden of depressive symptoms is observed among medical students throughout Pakistan. The results of the subgroup analysis are presented in table 1, which summarises the prevalence of depression across studies according to their geographical location.

**Table 1:** Subgroup analysis of depression prevalence by province

Province	Number of studies	Prevalence range (%)	I² (%)	P value for heterogeneity
Sindh	20	9–92	99.4	<0.001
Punjab	37	10.9–94	99.1	<0.001
Other regions	12	~12–68	97.7	<0.001
Overall	71	9–94	99.15	<0.001

### Subgroup analysis by assessment instrument

In this systematic review and meta-analysis, a subgroup analysis was conducted based on the depression assessment instruments used in the studies. The studies were divided into three subgroups^1^: DASS-21,^2^ PHQ-9 and^3^ other instruments (including BDI-I, BDI-II, HADS, CES-D10, ZUNG and others).

For studies using the DASS-21, prevalence estimates varied widely, ranging from approximately 33% to 94%, with a high level of heterogeneity (I²=97.6%, p<0.001). In the PHQ-9 subgroup, reported prevalence ranged from about 34.5% to 92%, also showing substantial heterogeneity (I²=98.9%, p<0.001).

For studies using other instruments, prevalence estimates were generally lower but still variable, ranging from approximately 9% to 89.3%, with high heterogeneity (I²=98.6%, p<0.001).

Overall, prevalence estimates across all studies ranged from 9% to 94%, indicating considerable variability. While differences between instrument subgroups were not statistically significant, studies using PHQ-9 and DASS-21 tended to report higher prevalence estimates compared with those using other instruments.

### Meta-regression analysis

A meta-regression analysis was conducted to investigate the potential impact of publication year on the prevalence of depressive symptoms among medical students in Pakistan.

**Figure 3:**
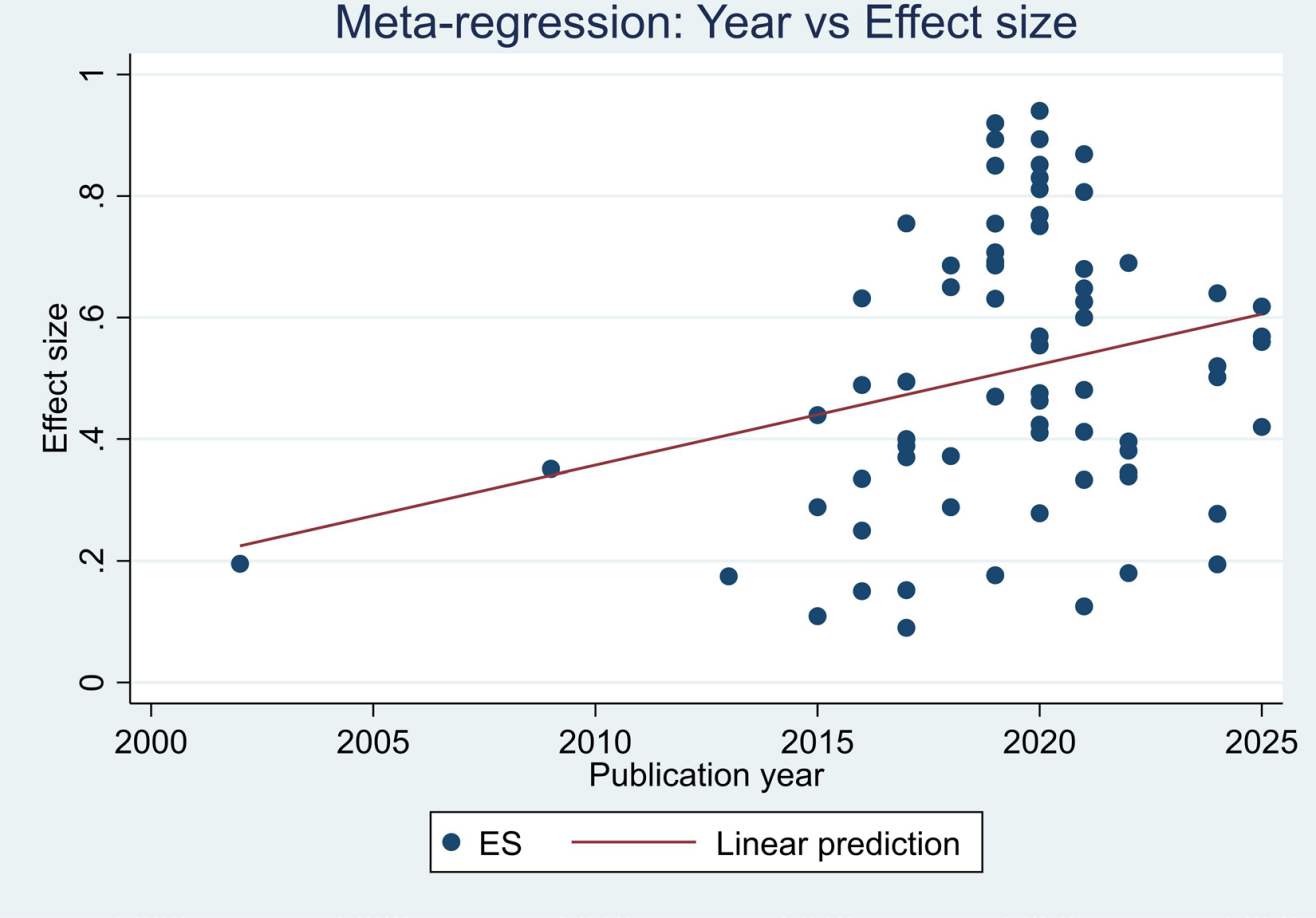
Bubble plot by publication year. ES, Effect Size.

In the initial model, there was no statistically significant association between publication year and depression prevalence (β=4.54, SE=4.14, 95% CI −3.58 to 12.65, p=0.27). However, in the final model, which considered the actual effect sizes, a significant positive correlation was found between the year of publication and the reported depression prevalence (β=0.016, SE=0.007, 95% CI 0.002 to 0.031, p=0.03). This suggests a gradual increase in the reported prevalence of depressive symptoms among medical students in Pakistan over time. The statistical model explained around 5% of the between-study variability, with a high residual heterogeneity (I²=99.1%). Figure 3 displays the bubble plot depicting the relationship between publication year and depression prevalence.

### Publication bias

**Figure 4:**
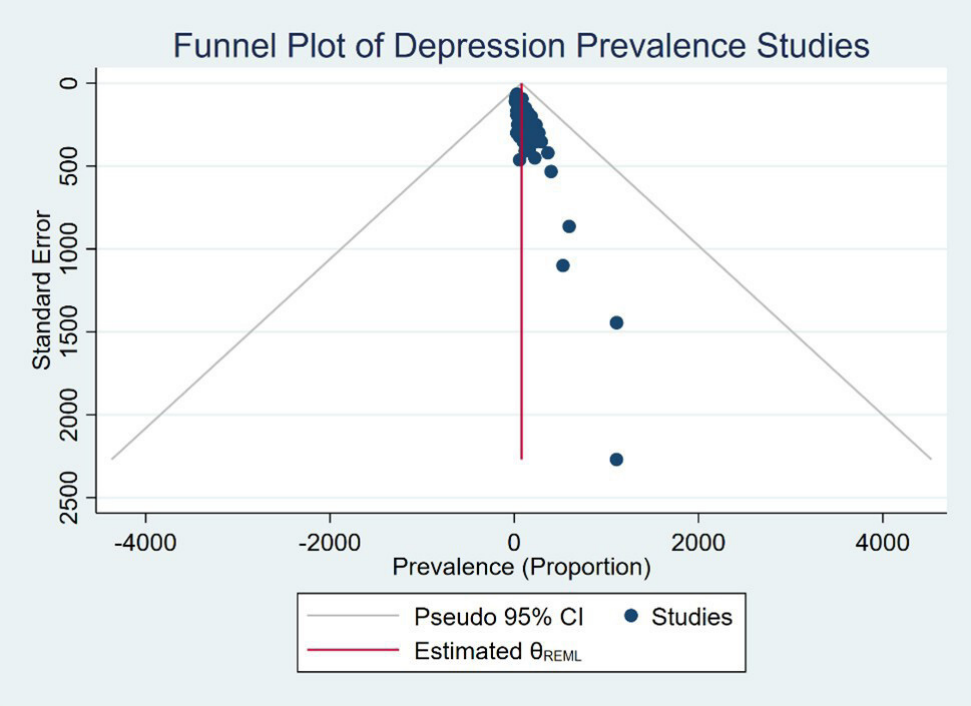
Funnel plot. REML, restricted maximum likelihood.

Publication bias was assessed using visual inspection of funnel plots, along with Egger’s and Begg’s tests. The results of Egger’s test showed a significant association between study size and effect size (β=0.52, SE=0.23, z=2.25, p=0.024), indicating potential publication bias. Begg’s test also supported these findings (z=4.67, p<0.001). The funnel plot (figure 4) showed some asymmetry, which is consistent with the statistical test results.

Although statistical tests suggested potential publication bias, trim-and-fill methods were not applied due to their limited reliability in the presence of substantial heterogeneity.

The methodological quality of the included studies was assessed using the JBI critical appraisal tool for analytical cross-sectional studies. Overall, the quality of studies varied considerably. Out of the included studies, approximately one-third were classified as high quality, while the majority were of moderate quality, and a smaller proportion were categorised as low quality. A detailed examination of individual domains revealed important sources of heterogeneity. In particular, issues related to sampling strategy were prominent. Although several studies reported the use of probability-based sampling methods (eg, simple or stratified random sampling), a large number of studies relied on non-probability or convenience sampling approaches. Furthermore, reporting of response rates was inconsistent across studies. Many studies did not report the number of participants approached or the response rate, making it difficult to assess the risk of non-response bias. Even among studies that claimed to use random sampling, insufficient reporting of recruitment procedures limited the evaluation of sample representativeness. In contrast, only a limited number of studies provided both a clear sampling strategy and an adequate response rate, allowing for a more reliable assessment of potential bias. These methodological differences, particularly in sampling and response reporting, contributed substantially to the observed heterogeneity across studies. The detailed results of the critical appraisal are presented in online supplemental table 2.

## Discussion

This systematic review and meta-analysis provides a comprehensive assessment of the prevalence of depression among medical students in Pakistan. The findings demonstrate a wide variation in reported prevalence estimates across studies, ranging from 9% to 94%, indicating substantial heterogeneity. This variability suggests that depressive symptoms are a significant mental health concern among medical students, although the burden may differ considerably depending on study context and methodology. These results align with data from various regions globally, indicating similarly elevated rates of depression among medical students. Rotenstein *et al* conducted a global meta-analysis and found that 27% of medical students experienced depression or depressive symptoms.^3^ Lin *et al* conducted a study during the COVID-19 pandemic and reported a prevalence of approximately 48%.^21^ A meta-analysis from Africa in 2024 found a prevalence of 38.8%,^22^ while a study in Saudi Arabia in 2020 reported a mean prevalence of 51.5%.^23^ The rise in depression rates among medical students in Pakistan can be attributed to a mix of personal, academic and environmental factors. Research in the country has indicated that factors like dissatisfaction with body image, academic pressure, conflicts with parents and instances of bullying are key predictors of depression in this group.^24^ Moreover, Fatimah *et al* found that financial issues (46.2%), heavy academic workload (26.1%), inadequate educational infrastructure like insufficient classroom space and facilities (9.4%), unsuitable dormitory environments (9.3%) and limited time for revision all play a role in causing depression among professional students, especially medical students. Furthermore, factors such as romantic disappointments, high self-expectations, intense academic competition and worries about future career prospects have also been consistently highlighted in previous research as significant contributors to depression in this group. The combination of these psychological, financial and academic pressures offers a plausible explanation for the high rates of depression seen in the current meta-analysis.^25^

The meta-regression findings showed a notable correlation between the year of publication and the prevalence of depression reported among medical students in Pakistan, suggesting a rise in depression rates over time. This increase may be attributed to various factors, including academic and financial stress, increased awareness and reporting of mental health concerns and the psychological effects of the COVID-19 pandemic. Similar trends of rising depression rates postpandemic have been observed in recent global studies involving medical students and healthcare professionals.^21^

The analysis showed a high level of variation among the studies included, likely due to differences in depression assessment tools, sample sizes and regional or cultural differences among medical students in Pakistan. Some of the variability and bias may be due to the deliberate exclusion of studies that reported depression alongside anxiety or other mental health conditions, especially those using local assessment tools like the Aga Khan University Anxiety and Depression Scale (AKUADS) that did not separate data for depression. Additionally, studies that reported combined prevalence rates for medical students, residents and general practitioners were excluded to maintain consistency in the target population.^26^ Despite these factors, the findings should be interpreted with caution given the substantial heterogeneity and potential publication bias across studies.

This review identified important methodological limitations across the included studies, particularly related to sampling strategies and response rate reporting, which are critical for prevalence studies. A substantial proportion of studies relied on non-probability or convenience sampling methods, including online surveys distributed via social media or institutional networks.^27^,^33^ Such approaches may introduce selection bias, as participants who are more engaged, accessible or interested in the topic are more likely to respond, potentially leading to overestimation or underestimation of prevalence. Even among studies that reported using random or probability-based sampling techniques,^34^,^37^ the lack of detailed information on the sampling frame, number of individuals approached and response rates limited the ability to assess the representativeness of the samples. This is particularly important in prevalence studies, where accurate estimation depends heavily on unbiased sampling. In addition, non-response bias could not be adequately evaluated in many studies due to the absence of response rate reporting.^2433 38^,^41^ Without this information, it remains unclear whether the included participants differ systematically from those who did not participate. In contrast, only a limited number of studies reported both probability-based sampling methods and adequate response rates,^35 36 42 43^ allowing for a more reliable assessment of potential bias. These findings highlight that, although the JBI tool provides an overall quality score, the risk of bias in prevalence studies is strongly influenced by the sampling methodology and participation rates. Therefore, future studies should prioritise probability-based sampling strategies and transparent reporting of recruitment processes and response rates to improve the validity and generalisability of findings.

### Limitations and recommendations

A key limitation of this systematic review was the lack of detailed data on the academic year of medical students in many of the included studies. Most studies reported the prevalence of depressive symptoms for the overall student population without distinguishing between different stages of training (eg, preclinical, clinical or internship). This limited our ability to explore potential variations across different phases of medical education.

Another important limitation relates to variability in the operational definition of depressive symptoms across studies. Although validated instruments were used, the cut-off thresholds applied were not consistent. In some studies, mild symptom categories were considered indicative of depressive symptoms, whereas others used higher thresholds reflecting more clinically significant symptoms. This inconsistency may have introduced misclassification and contributed to variability in prevalence estimates.

In addition, several studies did not clearly report key methodological details such as sampling frames, the number of questionnaires distributed or response rates, limiting the assessment of potential selection and non-response bias. These sampling-related limitations may have further contributed to the observed heterogeneity.

Importantly, the extremely high heterogeneity observed across studies substantially limits the interpretability and generalisability of the findings. Given this level of variability, pooled prevalence estimates may not accurately reflect the true burden across different settings and were, therefore, not emphasised in the analysis. In addition, prediction intervals were not calculated; however, the true prevalence is likely to vary widely across populations and contexts.

Finally, the absence of studies from Baluchistan province highlights a gap in the available evidence, possibly reflecting limited research infrastructure or under-representation of certain regions. Future research should adopt multicentre designs, report detailed methodological information (including sampling procedures and response rates) and apply standardised, clearly defined thresholds for depressive symptoms to improve comparability and strengthen the evidence base.

## Conclusion

The findings of this systematic review and meta-analysis highlight a substantial and concerning burden of depressive symptoms among medical students in Pakistan, with marked variability in prevalence estimates across studies. This aligns with global evidence, indicating that depression represents a significant challenge in medical education. However, the findings should be interpreted with caution due to the substantial heterogeneity across studies, variability in assessment instruments and cut-off thresholds, and methodological limitations related to sampling and response rate reporting. Despite these limitations, the observed variability and increasing trend over time underscore the urgent need for context-specific and scalable mental health interventions within academic settings. Medical universities should prioritise the implementation of mental health screening programmes, accessible counselling services and peer-support initiatives to facilitate early identification and management of psychological distress. Additionally, optimising academic workloads and restructuring clinical training environments may help reduce stress and improve students’ psychological well-being. A comprehensive approach that integrates individual, educational and institutional strategies is essential to foster a supportive learning environment and develop a resilient future healthcare workforce.

## Supplementary material

10.1136/bmjopen-2026-116544 online supplemental file 1

10.1136/bmjopen-2026-116544 online supplemental table 1

10.1136/bmjopen-2026-116544 online supplemental table 2

## Data Availability

All data relevant to the study are included in the article or uploaded as supplementary information.
