# Supplementary material for: Prevalence of depressive symptoms among medical students in Pakistan: a systematic review and meta-analysis
Source: BMJ Open. 2026 Jun 22;16(6):e116544. doi: 10.1136/bmjopen-2026-116544 (PMC13288679; doi:10.1136/bmjopen-2026-116544)
Supplement: online supplemental file 1 [file bmjopen-16-6-s001.docx]

**Search Strategy**

|  | **1. Ovid MEDLINE(R) ALL 1946 to September 29, 2025** |  |
| --- | --- | --- |
| 1 | exp Depression/ or exp Depressive Disorder/ or depress*.ti,ab. | 683141 |
| 2 | Students, Medical/ or exp Education, Medical/ or ((medic* and (student* or trainee* or learner*)) or resident* or "doctor* in training" or "physician* in training").ti,ab. | 516676 |
| 3 | exp Pakistan/ or Pakistan*.ti,ab,kf. | 43833 |
| 4 | 1 and 2 and 3 | **81** |
|  | **2. APA PsycInfo 1967 to September 2025 Week 3 (Ovid)** |  |
| 1 | exp "depression (emotion)"/ or depress*.ti,ab. | 385732 |
| 2 | medical students/ or exp medical education/ or ((medic* and (student* or trainee* or learner*)) or resident* or "doctor* in training" or "physician* in training").ti,ab. | 129693 |
| 3 | Pakistan*.mp. | 6331 |
| 4 | 1 and 2 and 3 | **27** |
|  | **3. Embase 1974 to 2025 Week 39 (Ovid)** |  |
| 1 | exp depression/ or depress*.ti,ab. | 1122580 |
| 2 | medical student/ or residency education/ or clinical education/ or ((medic* and (student* or trainee* or learner*)) or resident* or "doctor* in training" or "physician* in training").ti,ab. | 579842 |
| 3 | exp Pakistan/ or exp Pakistani/ or Pakistan*.ti,ab,kw. | 61949 |
| 4 | 1 and 2 and 3 | **192** |
|  | **4. Scopus** |  |
| #1 | TITLE-ABS-KEY (depress*) | 1,225,575 |
| #2 | TITLE-ABS-KEY((medic* AND (student* OR trainee* OR learner*)) OR resident* OR "doctor* in training" OR "physician* in training") | 903,149 |
| #3 | TITLE-ABS-KEY (Pakistan*) | 132,951 |
| #4 | #1 AND #2 AND #3 | **176** |
|  | **5. Web of Science Core Collection (SCIE, SSCI, and ESCI)** |  |
| #1 | TS= (depress*) | 958,693 |
| #2 | TS= ((medic* AND (student* OR trainee* OR learner*)) OR resident* OR "doctor* in training" OR "physician* in training") | 602,682 |
| #3 | TS= (Pakistan*) | 93,030 |
| #4 | #1 AND #2 AND #3 | **184** |
