# Supplementary material for: Prevalence of depressive symptoms among medical students in Pakistan: a systematic review and meta-analysis
Source: BMJ Open. 2026 Jun 22;16(6):e116544. doi: 10.1136/bmjopen-2026-116544 (PMC13288679; doi:10.1136/bmjopen-2026-116544)
Supplement: online supplemental table 1 [file bmjopen-16-6-s002.docx]

Table 1. Characteristics of the Included Studies

| Cut of Point | Tool | Female | Male | Prevalence | Sample  Size | SD | Mean | Author  Year  Location |
| --- | --- | --- | --- | --- | --- | --- | --- | --- |
| N/S^*^ | HADS | 87 | - | 19.5 | 87 | 1.9 | 20.7 | Rab[21]  2008  Punjab |
| BDI ≥14 | BDI | 106 | 27 | 35.1 | 279 | 1.41 | 21.4 | Alvi[22]  2009  Punjab |
| < 50 → Normal  50–59 → Mild depression  60–69 → Moderate depression  ≥ 70 → Severe depression | Zung SDS | - | - | 17.5 | 166 | - | - | Marwat[23]  2013  KPK |
| N/S | HADS | 55 | 55 | 10.9 | 110 | - | 21 | Khan[24]  2015  Punjab |
| score of ≥ 10 | DASS-42 | 38 | 28 | 44.3 | 66 | 1.3 | 22.1 | Rizvi[25]  2015  Islamabad and Punjab |
| cut-off score of ≥ 8 | HADS | - | - | 28.9 | 409 | 1.33 | 19.9 | Waqas[26]  2015  Punjab |
| ≥ 21Clinical Depression | BDI-II | 93 | 100 | 15 | 193 | - | - | Fatimah[27]  2016  Punjab |
|  | KADS-6 | 56 | 58 | 63.2 | 114 | - | - | Yousaf[28]  2016  Punjab |
| N/S | Self-reporting | 50 | 50 | 25 | 100 |  | - | Shafiullah[29]  2016  KPK |
| >4 indicates depression | QIDS-SR16 | 1235 | 1035 | 48.9 | 2270 | - | - | Shabbir[30]  2016  Sindh |
| Cut-off point: ≥14 | BDI-II | 128 | 72 | 33.5 | 200 | 1.9 | 21.8 | Ahsan[31]  2016  Punjab |
| N/S | BDI | 112 | 38 | 49.4 | 451 | 0.8 | 20.6 | Azad[32]  2017  Punjab |
| The cut off score was 7. The score of 0–7 was considered normal; a score of 8–10 considered borderline abnormal and score of 11–21 was considered case of a depression | HADS | 125 | 125 | 15.2 | 250 | NR | NR | Chaudhry[33] |
| N/S | DASS-21 | - | -- | 37~ | 100 | - | - | Naseem[34]  2017  Sindh |
| \| 0–4 No depression, 5–9 Mild, 10–14 Moderate, 15–19 Moderately Severe, 20–27 Severe \| \| --- \| | PHQ-9 | 147 | 53 | 75.5 | 200 | - | - | Uttra[35]  2017  Punjab |
| score of ≥ 10 | DASS-42 | 131 | 80 | 39 | 211 | 1.5 | 21.05 | Liaqat[36]  2017  Sindh |
| ≥ 20 = Clinical Depression | [BDI-II] | 218 | 82 | 9 | 300 | 2 | 23 | [Zafar](https://scholar.google.com/citations?user=GBejCVMAAAAJ&hl=en&oi=sra)[37]  2017  Sindh |
| < 50 → Normal  50–59 → Mild depression  60–69 → Moderate depression  ≥ 70 → Severe depression | Zung SDS | 184 | 186 | 40 | 370 | 3.19 | 23.05 | Mustafa[38]  2017  Punjab |
|  | DASS-21 | 135 | 75 | 68.6 | 210 | 1.78 | 21.84 | [39] Javaeed  2018  Kashmir |
|  | DASS-21 | 104 | 56 | 65 | 160 |  |  | Mughal[40]  2018  Islamabad |
|  | HADS | 253 | 156 | 28.9 | 409 | 1.33 | 19.86 | Waqas[41]  2018  Punjab |
|  | BDI | 183 | 67 | 37.2 | 250 | 1.58 | 20.97 | Afridi[42]  2018  kpk |
| cut-off ≥10 | PHQ-9 | 156 | 99 | 85 | 300 | - | - | Gitay[43]  2019  Sindh |
|  | DASS-21 | 88 | 100 | 71 | 188 | 2.2 | 21.4 | Azim[44]  2019  Sindh |
| cut-off ≥10 | PHQ-9 | 97 | 115 | 75.6 | 212 | - | 21 | Balouch[45]  2019  Sindh |
| PHQ-9 —  0–4 = none, 5–9 = mild, 10–14 = moderate, 15–19 = moderately severe, ≥20 = severe | PHQ-9 | 133 | 67 | 92 | 200 | 1.8 | 21.4 | Kumari[46]  2019  Sindh |
| N/S | Burn’s Depression | 176 | 124 | 89.3 | 300 | -- | - | Khan[47]  2019  Punjab |
| Normal: 0–9  Mild: 10–13  Moderate: 14–20  Severe: 21–27  Extremely Severe: ≥28 | DASS-21 | 135 | 75 | 68.6 | 210 | 1.7 | 21.8 | javaeed[48]  2019  Azad Jammu and Kashmir |
|  | DASS-21 | 244 | 111 | 63 | 355 | 3.2 | 22.7 | Akhtar Bibi[49]  2019  Pakistan |
| cut-off ≥10 | PHQ-9 | 95 | 85 | 47 | 183 | - | - | Majeed[50]  2019  Punjab |
|  | DASS-21 | 264 | 48 | 69.1^[[1]](#footnote-1)^ | 312 | 1.5 | 22.7 | Kumar[51]  2019  Sindh |
| < 50 → Normal  50–59 → Mild depression  60–69 → Moderate depression  ≥ 70 → Severe depression | Zung SDS | -- | - | 17.6 | 323 |  |  | Zafar[52]  2019  Punjab |
| PHQ-9 ≥ 10 | PHQ-9 | 1020 | 425 | 76.9 | 1445 | 3.1 | 21.8 | Ahmed[53]  2020  Sindh |
|  | DASS-21 | 116 | 66 | 85 | 182 |  |  | Ali[54]  2020  Sindh |
| < 50 → Normal  50–59 → Mild depression  60–69 → Moderate depression  ≥ 70 → Severe depression | Zung [SDS] | - | - | 41 | 402 | 1.83 | 21 | Qureshi[55]  2020  Sindh |
| N/S | PHQ-9 | 327 | 206 | 75 | 533 | 1.8 | 21.3 | Zafar[56]  2020  Punjab |
| Scores >25 | [BDI] | 240 | 76 | 27.84 | 316 | 4.03 | 21.48 | Zaidi[57]  2020  Sindh |
| PHQ-9 ≥ 5 | PHQ-9 | 199 | 154 | 83 | 353 | 1.5 | 20.5 | Waqas[58]  2020  Punjab |
| Cut-off point = BDI ≥ 17 | [BDI] | 83 | 148 | 55.4 | 231 | 2 | 21 | Ansar[59]  2020  KPK |
| PHQ-9 ≥ 5 | PHQ-9 | 1020 | 425 | 76.9 | 1445 | 3.1 | 21.8 | Ahmed[60]  2020  Sindh |
| Cut-off point = 10 | CESD-R-10 | 191 | 116 | 47.6 | 307 | 1.03 | 23.1 | Khan[61]  2020  Punjab |
| cut-off ≥10 | CESD-R-10 | 178 | 91 | 47.2 | 269 | 1.1 | 22.9 | Khan[62]  2020  Punjab |
| Normal = 0–9  Mild = 10–13  Moderate = 14–20  Severe = 21–Extremely Severe = ≥ 28 | DASS-21 | 156 | 125 | 94 | 251 | - | - | Ashraf[63]  2020  Punjab |
| Normal = 0–9  Mild = 10–13  Moderate = 14–20  Severe = 21–Extremely Severe = ≥ 28 | DASS-21 | 112 | 78 | 46.3 | 190 | 1.6 | 22.5 | Laique[64]  2020  Punjab |
| PHQ-9 ≥10 | PHQ-9 | 46 | 48 | 89.4 | 94 | - | - | Jafri[65]  2020  Sindh |
| Cut-off for presence of depression: ≥10 | DASS-21 |  |  | 81 | 233 | - |  | Masud[20]  2020  Punjab |
| N/S | HAM-D | 116 | 65 | 56.9 | 181 | 2.04 | 21.81 | [66] Siddiqui  2020  Sindh |
| N/S | DASS21 | 100 | - | 68 | 100 | 1.8 | 24.2 | Saghir[67]  2021  Punjab |
| Normal = 0–9  Mild = 10–13  Moderate = 14–20  Severe = 21–Extremely Severe = ≥ 28 | DASS-21 | 72 | 28 | 60.1 | 100 | - | - | Ashraf[68]  2021  Punjab |
| 0–4], mild [5–9], moderate [10–14], and severe15–21] depression | PHQ-9 | 756 | 344 | 48.1 | 1100 | 6 | 23.14 | Imran[69]  2021  Punjab |
| N/S | PHQ-9 | 184 | 236 | 86.9 | 420 | - | 21.7 | Kumar[70]  2021  Sindh |
| N/S | DASS-21 | 208 | 104 | 33.3 | 312 |  |  | Ahmed[71]  2021  Sindh |
| Cut-off point: Score ≥ 11 was considered as indicating depression | CES-D 10 | 265 | 198 | 12.5 | 463 | 1.6 | 20.2 | Rehman[72]  2021  Sindh |
| Cut-off for presence of depression: ≥10 | DASS-21 |  |  | 65 | 233 | - |  | Masud[20]  2021  Punjab |
| N/S | HADS | 109 | 41 | 80.7 | 150 | - | - | Ghani[73]  2021  Punjab |
| Mild = 10–13  Moderate = 14–20  Severe = 21–Extremely Severe = ≥ 28 | BDI-II | 151 | 99 | 41.2 | 250 | 2.1 | 21.4 | Anjum[74]  2021  Punjab |
| N/S | DASS-21 | 130 | 100 | 62.6 | 230 | 1.5 | 21.8 | Hassnain[75]  2021  Punjab |
| scores of 50–59 points MILD, 60–69 points Moderate, and 70 or more for sever | Zung SDS | NR | NR | 18 | 261 | NR | NR | Tahir[76]  2022  Punjab |
| 0–27 score [none: 0–4, mild: 5–9, moderate: 10–14, moderately severe: 15–19, severe: 20–27] | PHQ-9 | 366 | 498 | 69 | 864 | 1.3 | 19.8 | Khidri[77]  2022  Sindh |
| N/S | DASS | 145 | 105 | 39.6 | 250 | - | - | Amjad[78]  2022  Punjab |
| N/S | DASS-21 | 157 | 116 | 38 | 273 | - | 21.67 | Rizwan[79]  2022  Punjab |
| Normal = 0–9,  Mild = 10–13, Moderate = 14–20, Severe = 21–27, Extremely Severe = ≥28 | DASS-42 | 116 | 114 | 34 | 230 | - | - | Rajar[80]  2022  Sindh |
| No depression )0-4 points], mild depression [5-9], moderate depression [10-14], or severe depression [15-21 points] | PHQ-9 | 191 | 93 | 34.5 | 284 | - | - | Hyder Zaidi[81]  2022  Sindh |
| cut-off >10 | HADS | 172 | 151 | 19.4 | 324 | 1.6 | 21.7 | Gul[82] |
| N/S | DASS-21. |  |  | 64 | 100 |  |  | Iftikhar[83] |
| score was 9; 10–13 for mild depression, 14–20 for moderate depression, 21–27 was for severe depression, and 28+ for very severe depression | DASS-21 | 100 | - | 52 | 100 |  | 22.5 | Malik[84]  2024  Punjab |
| Scores 0 to 28 and above [normal [0-9], mild [10- 13], moderate [14-20], severe [21-27], extreme [>28] | DASS-21 | 291 | - | ≈ 50% | 291 | 1.7 | 21.4 | Siddique[85]  2024  Punjab |
| >3 out of 5 considered depressive symptoms | PHQ-2 | 255 | 98 | 27.8 | 353 | - | - | Sarwar[86]  2024  Punjab |
| PHQ-9 scores ≥10 | PHQ-9 | 140 | 94 | 56 | 234 | - | - | Usama[87]  2025  Punjab |
| N/S | DASS-21 | 156 | 98 | 61.9 | 254 | 1.66 | 21.6 | Ahmad[88]  2025  N/S |
| Normal [0–7], Mild [8–10], Moderate [11–14], Severe [≥15]. | HADS | 100 | 100 | 42 | 200 | - | - | Kiani[89]  2025  Islamabad |
| BDI > 10 | BDI | 93 | 67 | 57 | 160 | 1.1 | 20.5 | Muneeb[90]  2025  Sindh |

^*^N/S: Not specified, KPK: Khyber Pakhtunkhwa, MS: Medical student

| 21. Rab F, Mamdou R, Nasir S. Rates of depression and anxiety among female medical students in Pakistan. Eastern Mediterranean health journal = La revue de sante de la Mediterranee orientale = al-Majallah al-sihhiyah li-sharq al-mutawassit. 2008;14[1]:126-33. |  |
| --- | --- |
| 22. Alvi T, Assad F, Ramzan M, Khan FA. Depression, anxiety and their associated factors among medical students. Journal of the College of Physicians and Surgeons--Pakistan : JCPSP. 2010;20[2]:122-6. |  |
| 23. Marwat MA. Prevalence of depression and the use of antidepressants among third year medical students of khyber medical college, Peshawar. Journal of Postgraduate Medical Institute. 2013;27[1]:26 |  |
| EP - 8. |  |
| 24. Khan MA, Haider Z, Khokhar M. Anxiety and depression in 3rd year MBBS students of CMH ahore Medical College, Lahore, Pakistan. RAWAL MEDICAL JOURNAL. 2015;40[1]:21-3. |  |
| 25. Rizvi F, Qureshi A, Rajput AM, Afzal M. Prevalence of depression, anxiety and stress [by DASS scoring system] among medical students in Islamabad, Pakistan. Br J Med Med Res. 2015;8[1]:69-75. <https://doi.org/10.9734/BJMMR/2015/17193> |  |
| 26. Waqas A, Rehman A, Malik A, Muhammad U, Khan S, Mahmood N. Association of Ego Defense Mechanisms with Academic Performance, Anxiety and Depression in Medical Students: A Mixed Methods Study. Cureus. 2015;7[9]:e337. <https://doi.org/10.7759/cureus.337> |  |
| 27. Fatimah N, Hasnain Nadir M, Kamran M, Shakoor A, Mansoor Khosa M, Raza Wagha M. Depression among students of a professional degree: Case of undergraduate medical and engineering students. Int J Ment Health Psychiatry. 2016;2:2. |  |
| 28. Yousaf M, Daud S, Shafique MM. Gender Difference in Depression and Suicidal Ideation of medical students. PAKISTAN JOURNAL OF MEDICAL & HEALTH SCIENCES. 2016;10[3]:870-3. |  |
| 29. Shafiullah K, Khan S, Nisar S, Iftikhar ud D, Sadaf S. Prevalence of depression among students at Bacha Khan Medical College, Mardan. Journal of Medical Sciences [Peshawar]. 2016;24[4]:202 |  |
| EP - 5. |  |
| 30. Shabbir M, Bashir U. Depression among medical students. J Psychol Clin Psychiatry. 2016;6[5]:00371. <https://doi.org/10.15406/jpcpy.2016.06.00371> |  |
| 31. Ahsan U, Khan RMS, Latif A, Hussain S. Depression and its Associated Factors in Medical Students: A Cross Sectional Study. Pakistan Journal of Medical and Health Sciences. 2016;10[4]:1283-8. |  |
| 32. Azad N, Shahid A, Abbas N, Shaheen A, Munir N. Anxiety And Depression In Medical Students Of A Private Medical College. Journal of Ayub Medical College, Abbottabad : JAMC. 2017;29[1]:123-7. |  |
| 33. Chaudhry K, Ashraf M, Ibrahim M, Mahmood A, Zeb A. Prevalence of anxiety and depression among medical students of private medical college in Pakistan. Biomedica. 2017;33[2]:103-6. |  |
| 34. Naseem S, Munaf S. Suicidal Ideation, Depression, Anxiety, Stress, And Life Satisfaction Of Medical, Engineering, And Social Sciences Students. Journal of Ayub Medical College, Abbottabad : JAMC. 2017;29[3]:422-7. |  |
| 35. Uttra AM, Uttra MGM, Rauf A, Uttra MM, Hasan UH, Batool A. Prevalence of depression;: a cross-sectional study among mbbs Students of sargodha medical college, sargodha pakistan. The Professional Medical Journal. 2017;24[03]:482-9. <https://doi.org/10.29309/TPMJ/2017.24.03.1560> |  |
| 36. Liaqat H, Choudry U, Altaf A, Sauleh J, Rahman S, Choudry A, et al. Deranged mental homeostasis in medical students: evaluation of depression anxiety and stress among home and hostel students. Acta Psychopathol. 2017;3[1]:1-6. <https://doi.org/10.4172/2469-6676.100074> |  |
| 37. Zafar M, Rizvi SB, Sheikh L, Khalid Z, Abbas TG, Waseem S, et al. Comparative analysis of depression and its associated risk factors among public and private medical schools students in Karachi, Pakistan: a multicenter study. Saudi Journal for Health Sciences. 2017;6[1]:1-7. <https://doi.org/10.4103/sjhs.sjhs_83_16> |  |
| 38. Mustafa F, Muhammad A, Wajid S, Jalil F, Saleem M, Sadiq A. Depression among medical students and its association with gender, housing and year of study. Pakistan Journal of Medical and Health Sciences. 2020;14[1]:286 |  |
| EP - 9. |  |
| 39. Javaeed A, Zafar MB, Iqbal M, Ghauri SK. Correlation between internet addiction, depression, anxiety and stress among undergraduate medical students in Azad Kashmir. Pakistan Journal of Medical Sciences. 2019;35[2]:506 <https://doi.org/10.12669/pjms.35.2.169> |  |
| EP - 9. |  |
| 40. Mughal AM, Khokhar MM, Khokhor SM, Raja HB, Raja MU, Rashid A. Depression, Anxiety and Stress in Low and High Achieving Medical Students. Journal of Islamic International Medical College. 2019;14[3]:156-9. |  |
| 41. Waqas A, Naveed S, Aedma KK, Tariq M, Afzaal T. Exploring clusters of defense styles, psychiatric symptoms and academic achievements among medical students: a cross-sectional study in Pakistan. BMC research notes. 2018;11[1]:782. <https://doi.org/10.1186/s13104-018-3876-6> |  |
| 42. Afridi A, Nawaz Z, Asif S. Frequency of depression among medical students of medical colleges. Medical Forum Monthly. 2020;31[5]:74 |  |
| EP - 7. |  |
| 43. Gitay MN, Fatima S, Arshad S, Arshad B, Ehtesham A, Baig MA, et al. "Gender differences and prevalence of mental health problems in students of healthcare units": Correction. Community Mental Health Journal. 2019;55[5]:854. <https://doi.org/10.1007/s10597-018-0336-7> |  |
| 44. Azim SR, Baig M. Frequency and perceived causes of depression, anxiety and stress among medical students of a private medical institute in Karachi: a mixed method study. JPMA The Journal of the Pakistan Medical Association. 2019;69[6]:840-5. <https://doi.org/10.4135/9781529734348> |  |
| 45. Balouch MA, Anwar S, Ansari MI, Rasheed T, Ansari M, Balouch N. Impact of online social networking on mental health among medical students of Lumhs Jamshoro, Sindh, Pakistan. Rawal Medical Journal. 2019;44[3]:613 |  |
| EP - 7. |  |
| 46. Kumari U, Dawani N, Devnani J, Qureshi MFH, Soleja FK, Mohammad D, et al. Depression among medical students of Karachi a cross sectional study. MedEdPublish. 2019;8:181. <https://doi.org/10.15694/mep.2019.000181.1> |  |
| 47. Khan TA, Arif H, Sabahat S, Khan G, Khan A. Depression among Medical Students of a Public Sector Medical University in Pakistan. 2019. |  |
| 48. Javaeed A, Bint Zafar M, Iqbal M, Ghauri SK. Correlation between Internet addiction, depression, anxiety and stress among undergraduate medical students in Azad Kashmir. Pakistan journal of medical sciences. 2019;35[2]:506. <https://doi.org/10.12669/pjms.35.2.169> |  |
| 49. Bibi A, Blackwell SE, Margraf J. Mental health, suicidal ideation, and experience of bullying among university students in Pakistan. JOURNAL OF HEALTH PSYCHOLOGY. 2021;26[8]:1185-96. <https://doi.org/10.1177/1359105319869819> |  |
| 50. Majeed U, Sardar Z, Kiran N, Suqrat H, Sardar H, Adil S. Association of grit with depression among medical students. Annals of Punjab Medical College. 2019;13[4]:260-2. |  |
| 51. Kumar B, Shah MAA, Kumari R, Kumar A, Kumar J, Tahir A. Depression, Anxiety, and Stress Among Final-year Medical Students. Cureus. 2019;11[3]:e4257. <https://doi.org/10.7759/cureus.4257> |  |
| 52. Muhammad Alfareed Zafar S, Junaid Tahir M, Malik M, Irfan Malik M, Kamal Akhtar F, Ghazala R. Awareness, anxiety, and depression in healthcare professionals, medical students, and general population of Pakistan during COVID-19 Pandemic: A cross sectional online survey. Medical journal of the Islamic Republic of Iran. 2020;34:131. <https://doi.org/10.47176/mjiri.34.131> |  |
| 53. Ahmed M, Hamid R, Hussain G, Bux M, Ahmed N, Kumar M. Anxiety and depression in medical students of Sindh province during the covid-19 pandemic. Rawal Medical Journal. 2020;45[4]:947 |  |
| EP - 50. |  |
| 54. Ali A, Mahnoor S, Ahmed S, Naseem S, Shah SW, Shehryar S. COVID-19 Online Teaching and its Impact on Psychological Health in Higher Education: A Cross Sectional Study on Medical Students of 1st 2nd and 3rd Year MBBS. JOURNAL OF RESEARCH IN MEDICAL AND DENTAL SCIENCE. 2020;8[7]:275-9. |  |
| 55. Qureshi MFH, Mohammad D, Sadiq S, Abubaker ZJ, Kumari U, Devnani J, et al. A comparative cross-sectional analysis on prevalence of depression and associated risk factors among medical students and doctors of Karachi, Pakistan. Middle East Current Psychiatry. 2020;27[1]. <https://doi.org/10.1186/s43045-020-00066-5> |  |
| 56. Zafar U, Daud S, Khalid A. Determinants of depression among undergraduate medical students of a private medical college in Lahore. JPMA The Journal of the Pakistan Medical Association. 2020;70[3]:467-71. <https://doi.org/10.5455/JPMA.13896> |  |
| 57. Zaidi TH, Zafar M, Naz R, Farooq H, Khan D, Jawaid M, et al. Night eating syndrome among medical students and its correlation with depression in Karachi, Pakistan. Romanian Journal of Neurology/ Revista Romana de Neurologie. 2020;19[3]:193 <https://doi.org/10.37897/RJN.2020.3.9> |  |
| EP - 9. |  |
| 58. Waqas A, Iftikhar A, Malik Z, Aedma KK, Meraj H, Naveed S. Association of severity of depressive symptoms with sleep quality, social support and stress among Pakistani medical and dental students: A cross-sectional study. Global Psychiatry Archives. 2020;2[2]:211-20. <https://doi.org/10.52095/gpa.2020.1336> |  |
| 59. Ansar F, Ali W, Zareef A, Masud N, Zahab S, Iftekhar H. Internet addiction and its relationship with depression and academic performance: A cross-sectional study at a medical school in Pakistan. International Journal of Medical Students. 2020;8[3]:251-6. <https://doi.org/10.5195/ijms.2020.740> |  |
| 60. Ahmed M, Hamid R, Hussain G, Bux M, Ahmed N, Kumar M. Anxiety and depression in medical students of Sindh province during the Covid-19 pandemic. Rawal Medical Journal. 2020;45[4]:947-50. |  |
| 61. Khan TM, Bibi S, Shoaib E, Sufian HA, Dhillon AI, Mumtaz M. Association of depression with academic performance among Final Year MBBS students of Rawalpindi Medical University, Pakistan. European Journal of Medical and Health Sciences. 2020;2[6]. <https://doi.org/10.24018/ejmed.2020.2.6.529> |  |
| 62. Khan TM, Bibi S, Rasool ST, Jamil M, Khan S, Shafique H, et al. Impact of Depression on Food Consumption Frequency among Medical Students of Rawalpindi Medical University, Pakistan. European Journal of Medical and Health Sciences. 2020;2[6]. <https://doi.org/10.24018/ejmed.2020.2.6.578> |  |
| 63. Ashraf A, Ishfaq K, Ashraf MU, Ali J. Prevalence, Levels of Depression, Anxiety and Stress among Medical University Students [A Study of Nishtar Medical University Multan, Pakistan]. Journal of Languages, Culture and Civilization. 2020;2[2]:109-22. |  |
| 64. Laique T, Amin I, Jehangir MM, Hinamehmood, Rabiyasaif, Malik J. Integrated medical education system: Depression and anxiety among pakistani medical students. Pakistan Journal of Medical and Health Sciences. 2020;14[3]:694 |  |
| EP - 7. |  |
| 65. Jafri SR, Waseem U, Nazeer H, Muneeb A, Afzal MB, Qadir K. ASSESSMENT OF DEPRESSION AMONG UNIVERSITY STUDENTS USING PATIENT HEALTH QUESTIONNAIRE. Pakistan Armed Forces Medical Journal. 2020[5]:1443. |  |
| 66. Siddiqui NA, Fatima S, Taj FB, Shahid A, Moosa ZA. Depression among undergraduate medical and engineering students: A comparative study. Pakistan journal of medical sciences. 2020;36[5]:1096-9. <https://doi.org/10.12669/pjms.36.5.1858> |  |
| 67. Saghir M, Saleem MA, Kareem O, Riaz S, Majeed MK, Ramzan I. Depression, Anxiety and Stress among Medical and Non Medical Female Students of Pakistan. Pakistan Journal of Medical and Health Sciences. 2021;15[12]:3355 <https://doi.org/10.53350/pjmhs2115123355> |  |
| EP - 7. |  |
| 68. Ashraf MI, Suhail B, Saeed U, Arshad M, Imtiaz M, Zulqernain A. Psychiatric symptoms in students of medical colleges in Punjab. Pakistan Journal of Medical and Health Sciences. 2021;15[7]:1487 <https://doi.org/10.53350/pjmhs211571487> |  |
| EP - 90. |  |
| 69. Imran N, Haider II, Mustafa AB, Aamer I, Kamal Z, Rasool G, et al. The hidden crisis: COVID-19 and impact on mental health of medical students in Pakistan. Middle East Current Psychiatry. 2021;28[1]. <https://doi.org/10.1186/s43045-021-00123-7> |  |
| 70. Kumar R, Kumar H, Kumari R, Dars J, Qureshi S, Hamza MA, et al. The impact of covid-19 on medical students: A cross sectional survey. Pakistan Journal of Medical and Health Sciences. 2021;15[11]:2905 <https://doi.org/10.53350/pjmhs2115112905> |  |
| EP - 8. |  |
| 71. Ahmed FK, Sukhia H, Ejaz R, Khan QUA, Mushtaque U, Mushtaque S. Impact of COVID-19 on the Mental Health of Medical Students of Karachi, Pakistan. Pakistan Journal of Medical and Health Sciences. 2022;16[2]:903 <https://doi.org/10.53350/pjmhs22162903> |  |
| EP - 6. |  |
| 72. Rehman R, Fatima K, Hussain M, Sarim M, Gazzaz ZJ, Baig M. Association between depression and health risk behaviors among university students, Karachi, Pakistan. Cogent Psychology. 2021;8[1]:1886626. <https://doi.org/10.1080/23311908.2021.1886626> |  |
| 73. Ghani MU, Rana MM, Iqbal A, Fuaad M. Impact of social media on mental health among medical students of Private Medical College, Sargodha. Medical Forum Monthly. 2021;32[9]:163 |  |
| EP - 7. |  |
| 74. Anjum AF, Khokhar A, Ayaz H, Masud R, Rehman MU, Sadiq N, et al. Major predictor of depression among medical students: Body mass index. Pakistan Journal of Medical and Health Sciences. 2021;15[4]:790 |  |
| EP - 2. |  |
| 75. Hassnain S, Ahmad A, Qayyum MS, Farrukh MG, Nawaz UA, Ahmad H. Effects of covid-19 lockdown on mental health of medical students in lahore, Pakistan. Bangladesh Journal of Medical Science. 2021;20[5]:125 <https://doi.org/10.3329/bjms.v20i5.55406> |  |
| EP - 30. |  |
| 76. Junaid Tahir M, Tariq W, Anas Tahseen Asar M, Irfan Malik M, Kamal Akhtar F, Malik M, et al. Psychological Impact of COVID-19 on Doctors and Medical Students of Punjab, Pakistan: A Logistic Regression Analysis. Journal of multidisciplinary healthcare. 2022;15:1297-308. <https://doi.org/10.2147/JMDH.S369452> |  |
| 77. Khidri FF, Riaz H, Bhatti U, Shahani KA, Kamran Ali F, Effendi S, et al. Physical Activity, Dietary Habits and Factors Associated with Depression Among Medical Students of Sindh, Pakistan, During the COVID-19 Pandemic. Psychology research and behavior management. 2022;15:1311-23. <https://doi.org/10.2147/PRBM.S364540> |  |
| 78. Amjad N, Sarwar S, Khan MA, Sarrfraz F, Saeed R, Mehfooz Q. Relationship of Anxiety, Stress & Depression with Self Esteem among Undergraduate Medical Students. Pakistan Journal of Medical and Health Sciences. 2022;16[7]:400 <https://doi.org/10.53350/pjmhs22167400> |  |
| EP - 2. |  |
| 79. Rizwan HC, Ur Rahman S, Habib O, Khan MAW, Malik SB, Minhas IA. Relationship of Self-esteem with Depression, Anxiety and Stress among Pakistani medical students. Pakistan Journal of Medical and Health Sciences. 2022;16[2]:176 <https://doi.org/10.53350/pjmhs22162176> |  |
| EP - 8. |  |
| 80. Rajar AB, Channa NA, Mugheri MH. Self-Reported Depression and Its Relationship with Socio-Demographic Characteristics among Medical Students. Pakistan Journal of Medical and Health Sciences. 2022;16[8]:601 <https://doi.org/10.53350/pjmhs22168601> |  |
| EP - 4. |  |
| 81. Zaidi TH, Zafar M, Ilyas A, Khan M, Ghani R, Naz R, et al. Association of Burnout and Depression symptoms and their prevalence among medical students in Karachi, Pakistan. Russian Open Medical Journal. 2023;12[1]:e0104. <https://doi.org/10.15275/rusomj.2023.0104> |  |
| 82. Gul N, Ali A, Khan MS, Gul F, Gul A, Ali K, et al. Prioritizing Mental Health: A Cross-Sectional Investigation of Depression Prevalence and Risk Factors among Medical Students in Peshawar, Pakistan. International Journal of Medical Students. 2024;12[1]:22-8. <https://doi.org/10.5195/ijms.2024.2173> |  |
|  | |
| 83. Iftikhar N, Khaliq T. Comparison of Depression, Anxiety And Stress In Undergraduate And Postgraduate Medical Students. Pakistan Armed Forces Medical Journal. 2024;74[2]:300-3. <https://doi.org/10.51253/pafmj.v74i2.4162> |  |
| 84. Malik A, Bashir M, Lodhi FS, Jadoon ZG, Tauqir A, Khan MA. Depression, Anxiety and Stress using Depression, Anxiety, and Stress Scoring System [DASS-21] Among the Students of Women Medical and Dental College Abbottabad, Pakistan. Journal of Islamic International Medical College. 2024;19[2]:103-7. |  |
| 85. Siddique S, Atique H, Irfan A. Emotional state among medical students diagnosed with PCOS using the DASS 21. Rawal Medical Journal. 2024;49[1]:103 |  |
| EP - 6. |  |
| 86. Sarwar A, Waris H, Khan H, Umar MH, kumar Karmani V. Suicidal ideation, psychological distress and depression in medical students of Pakistan: Surviving or thriving. 2024. <https://doi.org/10.21203/rs.3.rs-3881550/v1> |  |
| 87. Usama M, Shahid S, Kamran MA, Rafiq MH, Khalid I, Mansoor MU. Investigating stress and depression among medical students: A cross-sectional study at Rawalpindi Medical University in Pakistan. 2025. <https://doi.org/10.5455/JPHCM.20241213012131> |  |
| 88. Ahmad A, Hassan Rizvi A, Uzair M, Bashir H, Amin M. Prevalence of internet addiction and its relation with depression, anxiety and stress in medical students of Pakistan. Psychology, health & medicine. 2025:1-10. <https://doi.org/10.1080/13548506.2025.2545020> |  |
| 89. Kiani MM, Khan S, Khursheed S, Kiani AI, Aftab A, Rahman A. Prevalence of Anxiety, Depression, Sleep Quality and Suicide Ideation among Medical Students in Islamabad, Pakistan. Journal of Bashir Institute of Health Sciences. 2025;6[1]:54-62. |  |
| 90. Muneeb A, Farrukh F, Nawaz B, Billia N, Fatima SE, Farooq M, et al. Frequency of Functional Depression and Coping Strategies in Medical Students and Doctors: Functional Depression and Coping Strategies. Pakistan BioMedical Journal. 2025:31-6. <https://doi.org/10.54393/pbmj.v8i2.1133> |  |

1. [↑](#footnote-ref-1)
