# Supplementary material for: Prevalence of depressive symptoms among medical students in Pakistan: a systematic review and meta-analysis
Source: BMJ Open. 2026 Jun 22;16(6):e116544. doi: 10.1136/bmjopen-2026-116544 (PMC13288679; doi:10.1136/bmjopen-2026-116544)
Supplement: online supplemental table 2 [file bmjopen-16-6-s003.docx]

Table .  Critical appraisal using the Analytical cross-sectional tool

| Citation | Q1 | Q2 | Q3 | Q4 | Q5 | Q6 | Q7 | Q8 | Q9 | quality | % |
| --- | --- | --- | --- | --- | --- | --- | --- | --- | --- | --- | --- |
| Rab et al | U | Y | N | Y | Y | Y | Y | U | U | M | 55.6 |
| Alvi | N | N | Y | Y | U | Y | Y | Y | U | M | 55.6 |
| Marwat | N | N | U | Y | U | Y | Y | Y | Y | M | 55.6 |
| Khan | N | N | U | Y | U | Y | Y | Y | U | L | 44.4 |
| Rizvi | N | N | N | Y | U | Y | Y | Y | U | L | 44.4 |
| Waqas | N | N | Y | Y | Y | Y | Y | Y | Y | H | 77.8 |
| Fatimah | U | Y | Y | Y | Y | Y | Y | Y | Y | H | 88.9 |
| Yousaf | N | N | N | Y | Y | Y | Y | Y | Y | M | 66.7 |
| Shafiullah | N | Y | N | Y | U | U | U | N | N | L | 33.3 |
| Shabbir | N | N | Y | Y | Y | Y | Y | Y | U | M | 66.7 |
| Ahsan | N | N | Y | Y | Y | Y | Y | Y | U | M | 66.7 |
| Azad | N | N | U | Y | Y | Y | Y | Y | U | M | 55.6 |
| Chaudhry | U | U | Y | Y | U | Y | U | Y | U | L | 44.4 |
| Naseem | N | N | Y | Y | Y | Y | Y | Y | U | M | 66.7 |
| Uttra | N | Y | Y | Y | Y | Y | Y | Y | Y | H | 88.9 |
| Liaqat | N | N | U | Y | Y | Y | Y | Y | U | M | 55.6 |
| Zafar | N | N | Y | Y | Y | Y | Y | Y | U | M | 66.7 |
| Mustafa | Y | Y | Y | Y | N | N | Y | Y | N/A | M | 66.7 |
| Javaeed | Y | Y | Y | Y | N | N | Y | Y | N/A | M | 66.7 |
| Mughal | Y | Y | Y | Y | N | N | Y | Y | N/A | M | 66.7 |
| Waqas | Y | Y | Y | Y | U | N | Y | Y | N/A | M | 66.7 |
| Afridi | Y | Y | Y | Y | Y | N | Y | Y | N/A | H | 77.8 |
| Gitay | Y | Y | Y | Y | Y | N | N | Y | N/A | M | 66.7 |
| Azim | Y | Y | Y | Y | Y | N | Y | Y | N/A | H | 77.8 |
| Balouch | Y | Y | Y | Y | U | N | Y | Y | N/A | M | 66.7 |
| Kumari | Y | Y | Y | Y | Y | N | Y | Y | N/A | H | 88.9 |
| Khan2019 | Y | Y | Y | Y | Y | N | Y | Y | N/A | H | 88.9 |
| Javaeed2019 | Y | Y | Y | Y | N | N | Y | Y | N/A | M | 66.7 |
| Bibi | Y | Y | Y | Y | Y | N | Y | Y | N/A | H | 88.9 |
| Majeed | Y | Y | Y | Y | U | Y | Y | Y | N/A | H | 88.9 |
| Kumar B | Y | Y | Y | Y | U | N | Y | Y | N/A | M | 66.7 |
| Muhammad A | Y | Y | Y | Y | Y | Y | Y | Y | N/A | H | 88.9 |
| Ahmed | Y | Y | Y | Y | Y | N | Y | Y | N/A | H | 77.8 |
| Ali A | Y | Y | Y | Y | U | N | Y | Y | N/A | M | 66.7 |
| Qureshi | Y | Y | Y | Y | Y | Y | Y | Y | N/A | H | 88.9 |
| Zafar U | Y | Y | Y | Y | Y | Y | Y | Y | N/A | H | 88.9 |
| Zaidi | Y | Y | Y | Y | U | N | Y | Y | N/A | M | 66.7 |
| Waqas A, Iftikhar A | Y | Y | Y | Y | Y | Y | Y | Y | N/A | H | 88.9 |
| Ansar | Y | Y | Y | Y | Y | Y | Y | Y | N/A | H | 88.9 |
| Ahmed M | Y | Y | Y | Y | Y | N | Y | Y | N/A | H | 77.8 |
| Khan TM | Y | Y | Y | Y | U | Y | Y | Y | N/A | H | 77.8 |
| Khan TM, Bibi S, Rasool ST | Y | Y | Y | Y | U | N | Y | Y | N/A | M | 66.7 |
| Ashraf | Y | Y | Y | Y | U | N | Y | Y | N/A | M | 66.7 |
| Laique | Y | Y | Y | Y | U | N | Y | Y | N/A | M | 66.7 |
| Jafri | Y | Y | Y | Y | U | N | Y | Y | N/A | M | 66.7 |
| Siddiqui | Y | Y | Y | Y | U | N | Y | Y | N/A | M | 66.7 |
| Saghir | Y | Y | Y | Y | U | N | Y | Y | N/A | M | 66.7 |
| Ashraf MI | Y | Y | Y | Y | U | N | Y | Y | N/A | M | 66.7 |
| Imran | Y | Y | Y | Y | Y | N | Y | Y | N/A | H | 77.8 |
| Kumar R, Kumar H | Y | Y | Y | Y | U | N | Y | Y | N/A | M | 66.7 |
| AHMED | Y | Y | Y | Y | U | N | Y | Y | N/A | M | 66.7 |
| Rehman | Y | N | Y | Y | Y | Y | Y | Y | N/A | H | 88.9 |
| Ghani | Y | N | U | Y | N | N | Y | Y | U | L | 44.4 |
| Anjum | Y | N | U | Y | N | N | Y | Y | U | L | 44.4 |
| Hassnain S | Y | Y | U | Y | N | N | Y | Y | U | M | 55.6 |
| Junaid | Y | N | Y | Y | Y | Y | Y | Y | Y | H | 88.9 |
| Khidri | Y | N | Y | Y | Y | Y | Y | Y | Y | H | 88.9 |
| Amjad | Y | N | U | Y | N | N | Y | Y | U | L | 44.4 |
| Rizwan | Y | N | U | Y | Y | Y | Y | Y | U | M | 66.7 |
| Rajar | Y | Y | U | Y | Y | Y | Y | Y | Y | H | 88.9 |
| Zaidi | Y | Y | Y | Y | Y | Y | Y | Y | U | H | 88.9 |
| Gul N | Y | Y | Y | Y | Y | Y | Y | Y | Y | H | 100 |
| Iftikhar | Y | N | Y | Y | Y | N | Y | Y | U | M | 66.7 |
| Malik | Y | Y | Y | Y | Y | N | Y | Y | Y | H | 88.9 |
| Siddique S | Y | Y | Y | Y | Y | N | Y | Y | U | H | 77.8 |
| Sarwar | Y | U | Y | Y | Y | Y | Y | Y | U | H | 77.8 |
| Ahmad A | Y | N | Y | Y | Y | Y | Y | Y | U | H | 77.8 |
| Kiani | Y | Y | Y | Y | Y | Y | Y | Y | U | H | 88.9 |
| Muneeb | Y | N | Y | Y | U | N | Y | Y | U | M | 55.6 |

Y: Yes. N: No, U: Unclear, L: Low, M: Moderate, H:High
