# Supplementary material for: Prevalence of depressive symptoms among medical students in Pakistan: a systematic review and meta-analysis
Source: BMJ Open. 2026 Jun 22;16(6):e116544. doi: 10.1136/bmjopen-2026-116544 (PMC13288679; doi:10.1136/bmjopen-2026-116544)
Supplement: Supplementary file 4 [file bmjopen-2026-116544.reviewer_comments.pdf]

PEER REVIEW HISTORY

BMJ Open publishes all reviews undertaken for accepted manuscripts. Reviewers are asked to complete a checklist review form and are provided with free text boxes to elaborate on their assessment. These free text comments are reproduced below.

ARTICLE DETAILS

Title (Provisional)

Prevalence of Depressive Symptoms Among Medical Students in Pakistan: A Systematic Review and Meta-Analysis

Authors

Ebrahimi, Hamideh; Bibi, Rashida; Falahatpisheh , Mohammad Hossein; Vedithi, Dr Sundeep Chaitanya

---

VERSION 1 - REVIEW

**Reviewer**	**1**
**Name**	**Mejia, Christian R.**
**Affiliation**	**Universidad Norbert Wiener**
**Date**	**29-Jan-2026**
**COI**	**None**

This statement raises some concerns: Assessments that reached or exceeded the threshold for mild depression were considered indicative of depression. If this were the case across all instruments, then it would be acceptable to analyze it accordingly. However, if this wasn't the case in other instruments/studies, it could lead to inflated results. Be very careful and thoroughly evaluate these cases, as they could introduce significant bias. This is discussed to some extent in the limitations section, but it should be examined in more detail in both the methodology and limitations sections, which I believe is the study's biggest problem.

The second paragraph of the introduction mentions that numerous studies show a higher prevalence in South Asia, but these article numbers are not cited. At least three or four should be cited, or the sentence should be revised.

The final paragraph of the introduction should also include citations, as it refers once again to numerous studies and various problems. It is recommended that each study be cited. The Burns Depression Inventory lacks references, unlike the others, so it would be preferable for this one to have them as well. In the introduction, some references are outdated. A thorough search would be ideal, as some phrases suggest the current situation, but the references are from the past (not all phrases, but some). The formatting of the references should also be reviewed; not all are following the Vancouver style.

**Reviewer**	**2**
**Name**	**Nikolakopoulos , Stavros**
**Affiliation**	**University of Ioannina**
**Date**	**10-Feb-2026**
**COI**	**None**

I would like to congratulate the authors on their excellent work. I greatly enjoyed reading the manuscript.

The present meta-analysis complements existing meta-analyses conducted in the field across different countries.

Major comments:

Page 6, Data analysis

The description of the statistical analysis methodology does not clearly specify the sensitivity analyses that were conducted to explore heterogeneity. In addition, the methodology does not take into account the computation of prediction intervals.

Page 6, line 26: Regarding the funnel plots, the trim-and-fill method was selected. However, this method is not recommended due to its poor performance in the presence of heterogeneity (1). Meta-analyses of proportions in general—and as also demonstrated by the results of the present meta-analysis—are typically characterized by substantial heterogeneity.

Page 7, line 17: The choice of a random-effects model is appropriate and recommended. Nevertheless, the method selected for transforming proportions in order to conduct the meta-analysis of proportions is not reported.

In addition, prediction intervals were not calculated. Given the extremely high heterogeneity observed (I^2 = 99.15%), the prediction interval would be expected to be very wide, potentially covering a large part of the 0–1 range. Therefore, although the pooled estimate suggests that the prevalence of depression in this population is 51%, the very high heterogeneity substantially limits the interpretability and generalizability of this finding. This constitutes a potentially important limitation of the study.

VERSION 1 - AUTHOR RESPONSE

Reviewer: 1
Dr. Christian R. Mejia, Universidad Norbert Wiener
Comments to the Author:
This statement raises some concerns: Assessments that reached or exceeded the threshold for mild depression were considered indicative of depression. If this were the case across all

instruments, then it would be acceptable to analyze it accordingly. However, if this wasn't the case in other instruments/studies, it could lead to inflated results. Be very careful and thoroughly evaluate these cases, as they could introduce significant bias. This is discussed to some extent in the limitations section, but it should be examined in more detail in both the methodology and limitations sections, which I believe is the study's biggest problem.

Response: We agree that variation in the definition of depression across studies and instruments is a critical methodological issue and may introduce bias if not properly addressed.In this study, we did not apply a uniform definition of depression across all instruments. Instead, prevalence estimates were extracted and reported according to the definitions and cut-off thresholds used in each individual study. This approach was adopted to preserve the original study-level classifications and avoid imposing arbitrary or inconsistent criteria across different instruments. We have revised the Methods section to clarify this approach more explicitly and to indicate that prevalence estimates were interpreted with reference to the validated thresholds of each instrument. We have also expanded the Limitations section to acknowledge that differences in operational definitions—particularly regarding whether mild symptom thresholds were considered indicative of depression—may have introduced variability and potential overestimation in some studies.

The second paragraph of the introduction mentions that numerous studies show a higher prevalence in South Asia, but these article numbers are not cited. At least three or four should be cited, or the sentence should be revised.
Response: Thank you for this helpful comment. The second paragraph of the Introduction has been revised. The previous statement referring to South Asia has been removed, and the text has been restructured to provide better-supported global and regional context. Relevant and appropriately cited studies have been incorporated to support statements regarding the prevalence of depressive symptoms among medical students.

The final paragraph of the introduction should also include citations, as it refers once again to numerous studies and various problems. It is recommended that each study be cited. The Burns Depression Inventory lacks references, unlike the others, so it would be preferable for this one to have them as well. In the introduction, some references are outdated. A thorough search would be ideal, as some phrases suggest the current situation, but the references are from the past (not all phrases, but some). The formatting of the references should also be reviewed; not all are following the Vancouver style.
Response: The Introduction section has been thoroughly revised to improve clarity, accuracy, and referencing. Relevant and up-to-date studies have been incorporated to appropriately support the statements, and outdated references have been updated where necessary and a reference has now been included for the Burns Depression Inventory. The reference list has also been carefully reviewed and reformatted according to the Vancouver style, and DOI numbers have been added where available.

Reviewer: 2

Dr. Stavros Nikolakopoulos , University of Ioannina
Comments to the Author:
I would like to congratulate the authors on their excellent work. I greatly enjoyed reading the

manuscript.
The present meta-analysis complements existing meta-analyses conducted in the field across different countries.

Major comments:
Page 6, Data analysis
The description of the statistical analysis methodology does not clearly specify the sensitivity analyses that were conducted to explore heterogeneity. In addition, the methodology does not take into account the computation of prediction intervals.

Response: Thank you for this important and constructive comment. In response to reviewer recommendations, the sensitivity analysis (leave-one-out approach) has been removed from the revised manuscript, as it was considered not informative given the substantial heterogeneity across studies and the limited interpretability of a single pooled estimate. We have also revised the Data Analysis section to clarify the analytical approach and to emphasize alternative methods used to explore heterogeneity, including subgroup analyses and meta-regression. Regarding prediction intervals, we acknowledge that they can provide useful information about the expected range of effects in future studies, particularly in the presence of high heterogeneity. However, given the very high between-study variability and our decision to de-emphasize pooled estimates, we focused on presenting the range of prevalence estimates and exploring variability across studies rather than relying on summary measures. We believe this approach offers a more transparent and informative representation of the data.

Page 6, line 26: Regarding the funnel plots, the trim-and-fill method was selected.

However, this method is not recommended due to its poor performance in the presence of heterogeneity (1). Meta-analyses of proportions in general—and as also demonstrated by the results of the present meta-analysis—are typically characterized by substantial heterogeneity.

Response: Thank you for this important and insightful comment. We agree that the trim-and-fill method may perform poorly in the presence of substantial heterogeneity, which is a key characteristic of the present meta-analysis (I^2 = 99.15%).In response, we have removed the trim-and-fill analysis, including the corresponding text and any related results, from the revised manuscript. Publication bias is now assessed using visual inspection of funnel plots along with Egger's and Begg's tests. We have also revised the relevant sections of the Methods and Results to reflect this change. Additionally, a clarification has been included in the manuscript to indicate that trim-and-fill methods were not applied due to their limited reliability under conditions of high heterogeneity.

Page 7, line 17: The choice of a random-effects model is appropriate and recommended. Nevertheless, the method selected for transforming proportions in order to conduct the meta-analysis of proportions is not reported.
In addition, prediction intervals were not calculated. Given the extremely high heterogeneity observed (I^2 = 99.15%), the prediction interval would be expected to be very wide, potentially covering a large part of the 0–1 range. Therefore, although the pooled estimate suggests that the prevalence of depression in this population is 51%, the very high heterogeneity substantially limits the interpretability and generalizability of this finding. This constitutes a

potentially important limitation of the study.

Response: We thank the reviewer for this important and constructive comment. We have clarified the method used for transforming proportions in the Methods section. Specifically, proportions were transformed using the Freeman–Tukey double arcsine transformation to stabilize variance.

In addition, we agree that the very high heterogeneity substantially limits the interpretability and generalizability of the findings. In the revised manuscript, we have avoided emphasizing pooled prevalence estimates and have explicitly acknowledged this issue as a key limitation in the Discussion section.

Given that pooled estimates were not emphasized in the revised analysis, prediction intervals were not calculated. Instead, we have highlighted the wide variability in prevalence estimates across studies and its implications for interpretation.

These revisions have been incorporated into the manuscript (Methods and Discussion sections).

Reviewer: 1
If you have selected 'Yes' above, please provide details of any competing interests.: N/A

Reviewer: 2
If you have selected 'Yes' above, please provide details of any competing interests.: Not applicable

VERSION 2 - REVIEW

**Reviewer**	**1**
**Name**	**Mejia, Christian R.**
**Affiliation**	**Universidad Norbert Wiener**
**Date**	**29-Mar-2026**
**COI**	

I can see that appropriate changes were made; consider it ready for publication.

VERSION 2 - AUTHOR RESPONSE

Thank you for this comment. Appropriate formatting amendments were made throughout the manuscript where applicable to improve consistency and readability.

Reviewer: 1
Dr. Christian R. Mejia, Universidad Norbert Wiener
Comments to the Author:
I can see that appropriate changes were made; consider it ready for publication.

Reviewer: 1
If you have selected ‘Yes’ above, please provide details of any competing interests.: Not applicable
